# Curcumin Inhibits Replication of Human Parainfluenza Virus Type 3 by Affecting Viral Inclusion Body Formation

**DOI:** 10.1155/2021/1807293

**Published:** 2021-08-09

**Authors:** Chaoliang Zhang, Kehan Zhang, Guangchao Zang, Tingting Chen, Nan Lu, Siyuan Wang, Guangyuan Zhang

**Affiliations:** ^1^Pathogen Biology and Immunology Laboratory and Laboratory of Tissue and Cell Biology, Experimental Teaching and Management Center, Chongqing Medical University, Chongqing 401331, China; ^2^Department of the First Clinical Medicine, Chongqing Medical University, Chongqing 401331, China; ^3^Department of Pathogen Biology, School of Basic Medicine, Chongqing Medical University, Chongqing 401331, China

## Abstract

Human Parainfluenza Virus Type 3 (HPIV3) is one of the main pathogens that cause acute lower respiratory tract infections in infants and young children. However, there are currently no effective antiviral drugs and vaccines. Herein, we found that a natural compound, curcumin, inhibits HPIV3 infection and has antiviral effects on entry and replication of the virus life cycle. Immunofluorescence and western blotting experiments revealed that curcumin disrupts F-actin and inhibits viral inclusion body (IB) formation, thus inhibiting virus replication. Curcumin can also downregulate cellular PI4KB and interrupt its colocalization in viral IBs. This study verified the antiviral ability of curcumin on HPIV3 infection and preliminarily elucidated its influence on viral replication, providing a theoretical basis for antiviral drug development of HPIV3 and other parainfluenza viruses.

## 1. Introduction

HPIVs are the second leading cause of acute lower respiratory tract disease in infants, following respiratory syncytial virus (RSV). Of the four serotypes of HPIVs, type 3 (HPIV3) is the most virulent and accounts for 60-70% of primary HPIV infection [1]. HPIV3 is a single-stranded, negative-sense, and nonsegmented enveloped RNA virus that mainly infects children under five years old, especially infants and young children around 1 year old. HPIV3 causes acute lower respiratory tract infection and induces bronchiolitis, pneumonia, etc. [2] and can also infect people with low immunity and cause upper respiratory tract infections [3]. In developed countries, the annual number of hospitalized patients infected with HPIV3 is as high as tens of thousands [4], and in developing countries, the lower respiratory tract caused by HPIV3 infection is also one of the important causes of infant death. To prevent and treat HPIV3 infection, there are currently no effective vaccines and antiviral drugs.

The genome of HPIV3 contains 15,462 nucleotides, encoding six main structural proteins [5]: nucleocapsid protein (N), phosphoprotein (P), RNA-dependent RNA polymerase large protein (L), matrix protein (M), and two surface glycoproteins hemagglutinin-neuraminidase protein (HN) and fusion protein (F) [6, 7]. After invading host cells, HPIV3 relies on N and P proteins to form viral inclusion bodies (IBs) in the host cell's cytoplasm. IBs are considered to be the center in which viral RNAs are synthesized [8, 9]. The formation of IBs requires not only viral proteins but also the assistance of host proteins. Zhang et al. found that acetylated *α*-tubulin plays a vital role in promoting the fusion of small IBs to form larger functional IBs [10]. F-actin and its regulatory factor cofilin also played a crucial role in forming HPIV3 IBs and synthesizing viral RNA [11]. According to reports, F-actin polymerization is closely related to the expression level of phosphatidylinositol-4-phosphate (PI4P) in cells. Since PI4P contributes to nucleation and comets formation of F-actin, PI4P must be induced by phosphatidylinositol 4-kinase beta (PI4KB) whose downregulation seriously affects the PI4P level in cells [12]. Recent research has shown that PI4KB can be recruited to replication sites via its interaction with viral proteins. In HPIV3- or HRSV-infected host cells, P protein of HPIV3 or N protein of HRSV can both recruit PI4KB to viral IBs, thus creating a PI4P-enriched microenvironment that reinforces IB structures and facilitates virus replication [13].

Curcumin is a natural phenolic compound extracted from a plant turmeric rhizome. It is widely used as a spice and coloring agent [14]. Simultaneously, it has a wide range of anti-inflammatory, antioxidant, and antitumor properties [15–17]. Recent studies have found that curcumin has broad-spectrum antiviral capabilities. Mazumder discovered that curcumin could inhibit HIV-1 integrase, thereby inhibiting HIV-1 infection [18], and inhibit HCV replication by interfering with the Akt-SREBP-1 pathway [19]. Curcumin can also interfere with binding of Zika and Chikungunya viruses to host cells to inhibit viral infection [20]. To inhibit hepatitis B virus, curcumin can downregulate the metabolic molecule PGC-1 [21]. Although curcumin pretreatment does not affect plaque formation of enterovirus 71 (EV71) [22], curcumin downregulates PI4KB expression in cells and affects the in vitro replication of EV71 [23]. Moreover, many studies have modified curcumin on silver, graphene, and other nanomaterials, and the composite nanomaterials demonstrated good antiviral activity and lowered biological toxicity against RSV infection [24, 25]. Several studies have shown that curcumin has antiviral ability against enveloped and nonenveloped viruses, and the antiviral mechanisms in different viruses are also diverse. However, the antiviral effect of curcumin on HPIV3 has been rarely reported.

Herein, we verified the antiviral ability of curcumin against HPIV3 and preliminarily clarified that curcumin can inhibit IB formation by disrupting F-actin's integrity. Simultaneously, curcumin can downregulate endogenous PI4KB level in cells, interfering with colocalization of PI4KB and IBs to affect IB formation, thereby inhibiting viral replication. The results of this study theoretically guide the development of new drugs against HPIV3 infection.

## 2. Materials and Methods

### 2.1. Cells, Viruses, Plasmids, and Reagents

A549, MDCK, and HeLa cells were cultured in Dulbecco's modified Eagle's medium (DMEM, Hyclone) supplemented with 16% fetal bovine serum (FBS, EverGreen), 1% penicillin, and streptomycin at 37°C with 5% CO_2_. HPIV3 (NIH47885) and recombinant HPIV3_HA-P_ were kindly granted by Professor Mingzhou Chen of Wuhan University and propagated in HeLa cells by inoculation at a multiplicity of infection (MOI) of 0.1. The plasmid pCAGGS-N-Flag encodes the HPIV3 N protein with a Flag tag at its C-terminus, and pCAGGS-HA-P encodes the HPIV3 P protein with an HA tag at its N-terminus [11]. Curcumin were purchased from Damas-beta and prepared into a storage solution of 10 mM.

### 2.2. Western Blot Analysis

Infected cells were washed, scraped into cold phosphate-buffered saline (PBS), pelleted by centrifugation at 13,000 rpm for 1 min, and then lysed in cold TNE buffer (50 mM Tris-Cl [pH 7.4], 150 mM NaCl, 2 mM EDTA [pH 8.0], 0.1% 2-mercaptoethanol, and protease inhibitor cocktail) for 30 min. Cell lysis was achieved through 30 rounds of Dounce homogenization on ice, and cell lysates were centrifuged at 13,000 rpm for 30 min at 4°C. The clarified supernatant was mixed with 5x SDS-PAGE loading buffer, boiled at 100°C for 10 min, and then subjected to 12% sodium dodecyl sulfate-polyacrylamide gel. The samples were then electroblotted onto a nitrocellulose membrane, blocked with skim milk in phosphate-buffered saline with Tween 20 (1/1,000 Tween 20) for 30 min at room temperature, and subsequently incubated with primary antibodies for 1 h and secondary antibodies for 45 min. The primary antibodies used were mouse anti-HPIV3 (1 : 2500, Abcam), rabbit anti-PI4KB (1 : 2500, Sigma), and rabbit anti-*β*-actin (1 : 1000, Proteintech). HRP-conjugated goat anti-mouse IgG (1 : 5000, Sigma) and HRP-conjugated goat anti-rabbit IgG (1 : 5000, Sigma) were employed as secondary antibodies.

### 2.3. Immunofluorescence Assay

HeLa cells grown on coverslips in 12-well plates were grown to 40–50% confluence and transfected or infected as indicated. After being harvested, the cells were washed three times with cold PBS, fixed with 4% paraformaldehyde for 20 min, permeabilized with 0.2% Triton X-100 for 20 min, and blocked by 3% bovine serum albumin (BSA) for 30 min, and the cells were then incubated with relative primary antibodies for 1.5 h at room temperature. The used primary antibodies include rabbit anti-*α*-tubulin, rabbit anti-PI4KB, and mouse anti-HA tag, depending on the situation. The cells were then washed three times with 1% BSA and incubated with relative secondary antibodies for 45 min at room temperature. The secondary antibodies used were Alexa Fluor 488-conjugated goat anti-rabbit IgG (1 : 1000, Thermo) and Alexa Fluor 568-conjugated goat anti-mouse IgG (1 : 1000, Thermo). F-actin was stained with Alexa Fluor 488-conjugated Phalloidin (1 : 1000, AAT Bioquest), and cell nuclei were stained with DAPI (SolarBio). Images were observed via an immunofluorescence microscope (Nikon Ts2-FL).

### 2.4. Virus Infection and Plaque Assay

Cells in 6-well plates were grown to 60–70% confluence and infected with wild-type HPIV3 or HPIV3_HA-P_ for 2 h at 37°C with 5% CO_2_, and the infection medium was then removed and changed with fresh medium containing 4% FBS. In the plaque assay, HPIV3-containing culture medium was serial 10-fold diluted up to 10^−5^. HeLa cells in 24-well plates were grown to 60-70% confluence and infected with 400 *μ*L dilutions. After incubation for 2 h at 37°C with 5% CO_2_, the infection medium was replaced with methylcellulose, and the cell plates were incubated at 37°C with 5% CO_2_ for additional 4–5 days until detecting visible viral plaques. Plates were stained with 0.5% crystal violet for at least 12 h at room temperature and washed, the plaques were then countered, and the viral titers were calculated.

### 2.5. Drug Treatment Assay

To investigate curcumin impact on different stages of the viral life cycle, three different treatment conditions were performed: (1) pretreatment group: A549 cells were treated with 20 *μ*M curcumin for 6 h, washed once with PBS, and followed by infecting with HPIV3 (MOI of 0.1) for 2 h; then, the infection medium was changed with fresh medium containing 4% FBS; (2) cotreatment group: A549 cells were infected with HPIV3 and simultaneously treated with 20 *μ*M curcumin; 2 h after infection, the infection medium was changed with fresh medium containing 4% FBS; and (3) posttreatment group: A549 cells were firstly infected with HPIV3 for 2 h without curcumin; then, the infection medium was changed with a fresh medium containing 4% FBS and 20 *μ*M curcumin.

### 2.6. Drug Addition and Removal Assay

A549 cells were infected with HPIV3 (MOI of 0.1) for 2 h at 37°C; the infection medium was replaced with a fresh medium containing 4% FBS and 20 *μ*M curcumin. The drug-containing medium was removed at 6 h, 12 h, 24 h, 36 h, and 48 h after treatment by washing cells once with PBS; then fresh medium containing 4% FBS was added.

### 2.7. Quantitative Real-Time PCR

Cellular total RNA was extracted from A549 cells with TRIzol reagent (Ambion) and reverse transcribed into cDNAs using the M-MLV reverse transcriptase (Promega). The quantities of HPIV3 viral RNAs and *β*-actin were quantified using SYBR Premix Ex Taq II (Takara) and a LightCycler (Roche). Data shown are the relative abundance of the viral RNAs normalized to that of *β*-actin. The primers were as follows: HPIV3 viral gene forward: 5′-GGACATTCATGCCCAGATG-3′; HPIV3 viral gene reverse: 5′-AGCTCGTTTACCCTTTCAG-3′.

### 2.8. Image and Statistical Analysis

The amounts of protein in cell lysates were estimated based on the density of protein bands using Bio-Rad software Quantity One-4.6.2, and the histograms for the western blot were derived as follows: the amount of HN or the PI4KB/*β*-actin amount in the corresponding lysate is normalized to the value obtained in the DMSO group, and the value is set to 1.0. The results are shown as average with standard deviation (SD), and GraphPad Prism 5.0 was used to analyze the statistics. Statistical analyses were performed using Student's *t*-test, and *p* values less than 0.05 were considered significantly different and indicated by asterisks in the figures.

## 3. Result

### 3.1. Curcumin Inhibits HPIV3 Infection in a Dose-Dependent Manner

**Figure 1:**
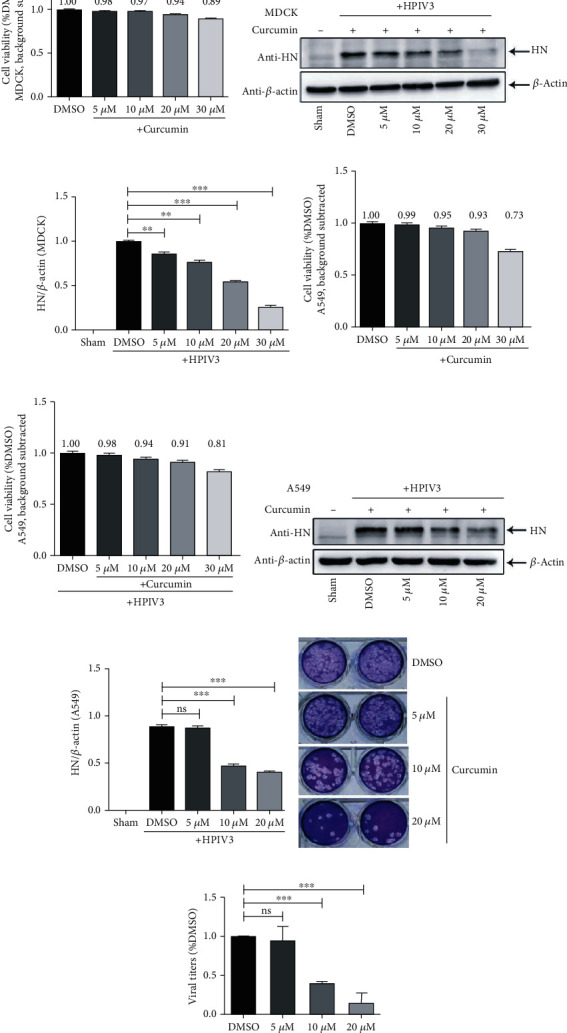
Curcumin inhibits HPIV3 infection. (a) MDCK cells were incubated with different concentrations of curcumin (5 *μ*M, 10 *μ*M, 20 *μ*M, and 30 *μ*M) or control DMSO for 48 h; the cell viability was examined by the CCK-8 assay as per the manufacturer's instruction. There was no significant toxicity when cells were treated with 5 *μ*M, 10 *μ*M, and 20 *μ*M curcumin. (b, c) MDCK cells were treated with the abovementioned concentrations of curcumin or DMSO for 6 h; then, cells were washed and infected by HPIV3 (MOI = 0.1) with different concentrations of curcumin present. The sham group was neither virus-infected nor drug-treated. Cells were collected after 36 h infection, viral HN protein was analyzed by western blot (WB), and cellular *β*-actin was used as a loading control. HN/*β*-actin for each group was calculated as described in Materials and Methods. Data are means ± SD from three experiments. Student's test: ^∗∗^*p* < 0.01 and ^∗∗∗^*p* < 0.001. A549 cells noninfected (d) or infected with HPIV3 (e) were incubated with different concentrations of curcumin (5 *μ*M, 10 *μ*M, 20 *μ*M, and 30 *μ*M) or control DMSO for 48 h; the cell viability was examined as above. (f, g) A549 cells were treated with curcumin and infected with HPIV3 as described in (b, c). Cells were collected after 36 h infection, viral HN protein and cellular *β*-actin were analyzed by WB, and HN/*β*-actin for each group was calculated as above. Data are means ± SD from three experiments. Student's test: ns: nonsignificant; ^∗∗∗^*p* < 0.001. (h, i) Viral plaques and titers in the culture medium of (f, g) were determined by plaque assay as described in Materials and Methods. Data are means ± SD from three experiments. Student's test: ns: nonsignificant; ^∗∗∗^*p* < 0.001.

Curcumin exhibits various biological activities, and to eliminate the influence of its antitumor effect, we first chose noncancer cell line MDCK cells to conduct antiviral experiments of curcumin against HPIV3. The cytotoxicity test was carried out in MDCK cells, which were incubated with curcumin (5 *μ*M, 10 *μ*M, 20 *μ*M, and 30 *μ*M) for 48 hours, and the cell viability was detected with the CCK-8 method using DMSO as the control group. The results showed that when treated with 30 *μ*M curcumin, the cell viability was still 89% (Figure 1(a)), indicating that the drug has no obvious toxic effect on cells. To verify the antiviral effect of curcumin on HPIV3, MDCK cells were treated with curcumin at the above concentrations for 6 h and were then infected by HPIV3. At 36 h infection, the cells were collected to detect the virus protein expression in cell lysates. The results revealed that compared with the DMSO treatment group, viral HN protein expression decreased with increasing curcumin concentration (Figures 1(b) and 1(c)). To test the effect of the antiviral compound curcumin on respiratory epithelial cells, curcumin toxicity assays were performed using A549 cells. When treated with 5, 10, or 20 *μ*M curcumin, no obvious toxic effect was observed in A549 cells with or without HPIV3 infection (Figures 1(d) and 1(e)). Next, cells were infected, then treated with curcumin, as described above. At 36 h postinfection, cells were collected, and viral protein expression was examined. Western blot results showed that treatment of infected cells with 10 or 20 *μ*M curcumin significantly reduced the HN protein expression in cell lysates (Figures 1(f) and 1(g)). Viral plaques and culture medium viral titers were also greatly reduced compared to those in the DMSO group (Figures 1(h) and 1(i)).

The above results indicate that curcumin can inhibit HPIV3 infection, and its inhibitory effect on the virus is dose-dependent.

### 3.2. Curcumin Inhibits HPIV3 Entry and Replication

**Figure 2:**
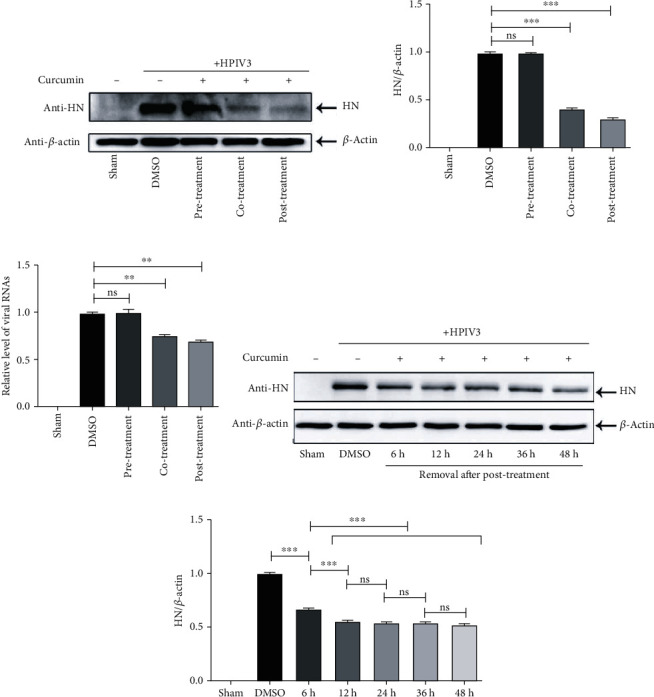
Curcumin inhibits multiple stages of the HPIV3 life cycle. (a, b) A549 cells were pretreated, cotreated, or posttreated with 20 *μ*M curcumin as described in Materials and Methods. The sham group was nontreated. Cells were collected after 36 h infection, and the viral HN protein and control *β*-actin were detected by WB. HN/*β*-actin for each group was calculated as described in the legend for Figure 1. Data are means ± SD from three experiments. Student's test: ns: nonsignificant; ^∗∗∗^*p* < 0.001. (c) Pre-, co-, or posttreated A549 cells were collected, and real-time PCR was performed as described in Materials and Methods to detect viral RNAs. Cellular *β*-actin mRNA was used as the control. Samples were examined in triplicate, and data are means ± SD from three experiments. Student's test: ns: nonsignificant; ^∗∗^*p* < 0.01. (d, e) A549 cells were infected with HPIV3 and posttreated with 20 *μ*M curcumin as above. Then, curcumin was removed after 6 h, 12 h, 24 h, 36 h, and 48 h of posttreatment. Cells were collected, and the viral HN protein and control *β*-actin were detected by WB. HN/*β*-actin for each group was calculated as above. Data are means ± SD from three experiments. Student's test: ns: nonsignificant; ^∗∗∗^*p* < 0.001.

To explore the specific stage of curcumin inhibiting HPIV3 infection, three different treatment conditions of A549 cells (pretreatment, cotreatment, and posttreatment) were performed as described in Materials and Methods, and viral proteins in different treatment groups were detected using WB. HN protein expression remained comparable between the pretreated and DMSO-treated groups, indicating that incubating cells in advance with the drug does not prevent HPIV3 infection. HN expression levels in cotreated and postinfection-treated cells were substantially decreased compared to DMSO treatment, suggesting that curcumin may block virus entry into host cells and may inhibit subsequent steps in virus replication and/or budding (Figures 2(a) and 2(b)). We also examined the level of viral RNA synthesis upon curcumin treatment. A549 cells were pre-, co-, or postinfection treated as described above, and real-time PCR assays were performed. Viral RNAs were suppressed by co- and postinfection treatments, but not preinfection treatment, compared to the DMSO group (Figure 2(c)). To explore in detail the effect of curcumin on the viral replication cycle, infected A549 cells were treated with curcumin, and the drug was removed at different time points (6, 12, 24, 36, and 48 h) after treatment. Cells were collected and viral proteins detected using WB. When the drug was removed at 6 h posttreatment, HN expression decreased relative to the DMSO group, indicating that curcumin inhibits virus entry. HN expression levels with drug removal at 12, 24, 36, and 48 h after treatment were almost equivalent to each other but were all lower than when the drug was removed at 6 h (Figures 2(d) and 2(e)), suggesting that curcumin inhibits subsequent viral replication steps but seems to exert no influence on virus viral budding. The above experiments show that curcumin inhibits HPIV3 infection mainly by interfering with virus entry and replication.

### 3.3. Curcumin Inhibits the Formation of Viral IBs

**Figure 3:**
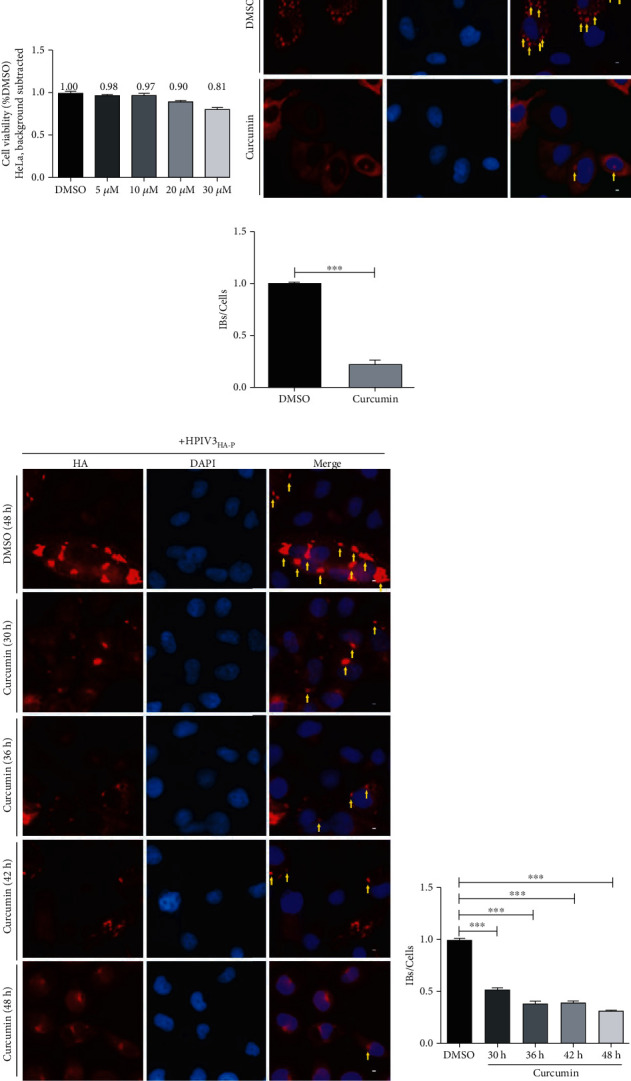
Curcumin inhibits the formation of viral IBs. (a) HeLa cells were incubated with curcumin or DMSO for 48 h, and the cell viability was examined as described above. There was no significant toxicity when cells were treated with 20 *μ*M curcumin. (b, c) HeLa cells were cotransfected with plasmids encoding N-Flag and HA-P jointly by lipo3000 as per the manufacturer's instruction. DMSO or curcumin were added at 12 h before sample collection. At 24 h posttransfection, cells were collected and fixed, mouse HA primary antibody and goat anti-mouse AF568 were used to stain P protein to visualize IBs, and nuclei were counterstained with DAPI. Scale bar = 10 *μ*m. The yellow arrow indicates IBs. Quantification for IBs is defined as the average number of IBs in each cell (IBs/cells), and IBs in at least 50 cells were calculated for each group. The histogram shows IBs/cells of the curcumin treatment group relative to the DMSO treatment group. Data are means ± SD from three experiments. Student's test: ^∗∗∗^*p* < 0.001. (d, e) HeLa cells were infected with HPIV3_HA-P_ at an MOI of 1.0, curcumin were added at 24 h after infection, and samples were collected at 30, 36, 42, and 48 h postinfection. Then, the cells were fixed as described above, HA-P was immunostained to visualize IBs, and nuclei were counterstained with DAPI. Scale bar = 10 *μ*m. The yellow arrow indicates IBs. Quantification for IBs was calculated as described above. Data are means ± SD from three experiments. Student's test: ^∗∗∗^*p* < 0.001.

The IBs of HPIV3 are composed of viral N and P proteins and related host proteins, which are considered as factories for viral replication. To explore the curcumin mechanism on the replication stage of HPIV3, we conducted immunofluorescence experiments in HeLa cells to verify whether curcumin affects viral IB formation. Before this, the CCK-8 method was employed to detect the cell viability of HeLa cells after curcumin incubation for 48 h. The results showed that when curcumin concentration was 20 *μ*M, the cell viability was 90%, and no obvious cytotoxic effect is found on HeLa cells (Figure 3(a)). Subsequently, the plasmids expressing viral proteins N-Flag and HA-P were cotransfected into HeLa cells by lipo3000, and 20 *μ*M curcumin was added at 12 h posttransfection; then, the samples were collected at 36 h after transfection for immunofluorescence experiments. The results showed that, compared with DMSO treatment, the number of dot-like IBs in each cell was reduced by nearly 90% after curcumin treatment (Figures 3(b) and 3(c)). To further verify curcumin impact on viral IBs regarding virus infection, we used a recombinant HPIV3 virus, i.e., HPIV3_HA-P_, with a HA tag fused to the N terminus of the P protein. HeLa cells were infected with HPIV3_HA-P_. At 24 h after infection, 20 *μ*M curcumin was added; then, the samples were collected at 30 h, 36 h, 42 h, and 48 h postinfection. The results of immunofluorescence experiments showed that after curcumin treatment for different times, syncytium in cells formed by HPIV3_HA-P_ infection was greatly decreased, indicating the inhibition effect of curcumin on virus infection. Consisting with transfection, the number of viral IBs in the infected cells was reduced by up to 75% after curcumin treatment (Figures 3(d) and 3(e)). It suggests that curcumin can affect viral IB formation and thus inhibit viral replication.

### 3.4. Curcumin Disrupts the Integrity of F-Actin and Affects the Formation of Viral IBs

**Figure 4:**
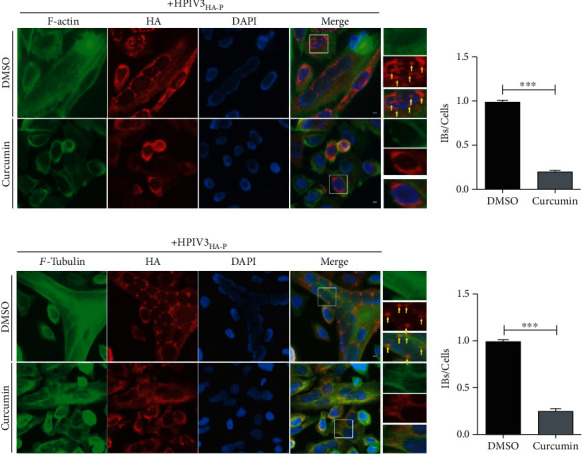
Curcumin damages the structural integrity of F-actin. (a, b) HeLa cells were infected with HPIV3_HA-P_ at an MOI of 1.0, DMSO and curcumin were added at 12 h before sample collection, and cells were collected and fixed 36 h after infection. F-actin was stained with phalloidin-AF488, HA-P was immunostained to visualize IBs, and nuclei were counterstained with DAPI. Scale bar = 10 *μ*m. The yellow arrow indicates IBs, and quantification for IBs was calculated as described in the legend for Figure 3. Data are means ± SD from three experiments. Student's test: ^∗∗∗^*p* < 0.001. (c, d) HeLa cells were infected with HPIV3_HA-P_ and treated as described in (a, b). At 36 h after infection, cellular microtubules were stained with rabbit anti-*α*-tubulin primary antibody and goat anti-rabbit AF488, HA-P was immunostained to visualize IBs, and nuclei were counterstained with DAPI. Scale bar = 10 *μ*m. The yellow arrow indicates IBs, and quantification for IBs was calculated as described above. Data are means ± SD from three experiments. Student's test: ^∗∗∗^*p* < 0.001.

Cytoskeleton destruction affects the transcription and replication of HPIV3 [26, 27]. During HPIV3 IB formation, a complete cytoskeleton is required as the platform. Therefore, curcumin impact on HPIV3 IBs may be due to the effect of drugs on the cytoskeleton. To verify the above speculation, HeLa cells were infected with HPIV3_HA-P_ for 24 h, then treated with 20 *μ*M curcumin. At 36 h postinfection, the cells were fixed, and the structures of *α*-tubulin and F-actin and IB formation were observed by immunofluorescence. The results showed that after curcumin treatment, the stress fibers of F-actin were severely damaged, and IB formation was significantly inhibited. Compared with the DMSO group, the number of IBs in curcumin-treated cells decreased by about 60% (Figures 4(a) and 4(b)). Although the *α*-tubulin structure did not seem to be affected by curcumin, IB formation is reduced by about 50% (Figures 4(c) and 4(d)). It indicates that the curcumin effect on IB formation is probably due to F-actin destruction.

### 3.5. Curcumin Downregulates PI4KB Expression and Interferes with Its Colocalization with IBs

**Figure 5:**
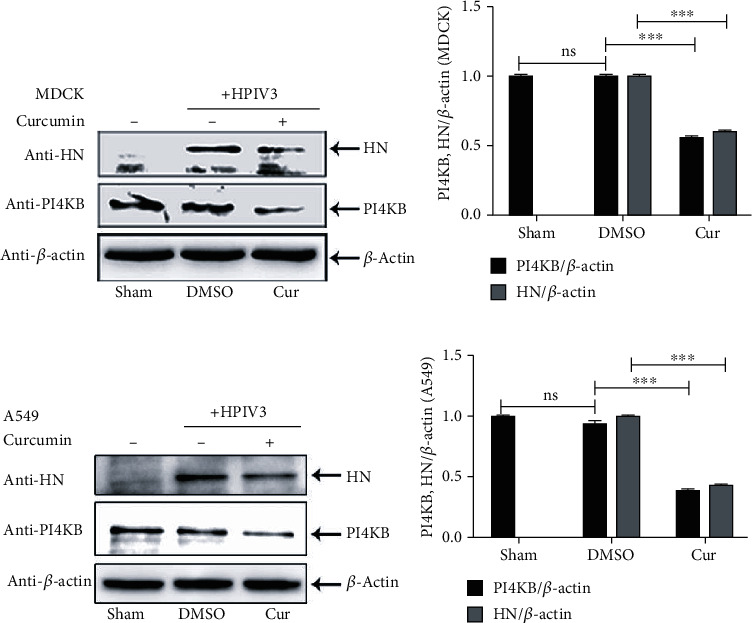
Curcumin downregulates cellular PI4KB. MDCK cells (a, b) and A549 cells (c, d) were infected with HPIV3 at an MOI of 0.1; DMSO or curcumin was added after infection. The sham group was nontreated. Cells were collected 36 h after infection, and the HN protein, PI4KB, and *β*-actin were detected by WB as described in the legend for Figure 1. PI4KB or HN/*β*-actin for each group was calculated as described in Materials and Methods. Data are means ± SD from three experiments. Student's test: ns: nonsignificant; ^∗∗^*p* < 0.01; ^∗∗∗^*p* < 0.001.

**Figure 6:**
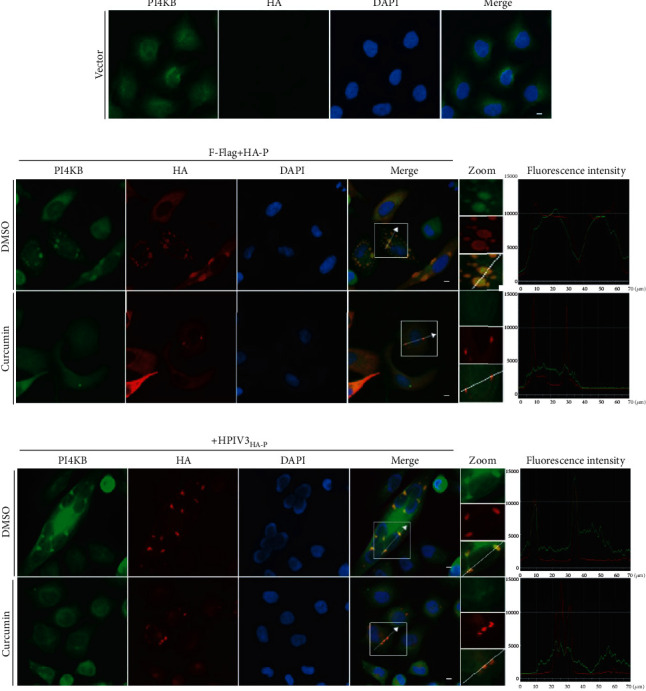
Curcumin interferes with colocalization of PI4KB in IBs. (a) HeLa cells were fixed, and cellular PI4KB was stained with rabbit anti-PI4KB primary antibody and goat anti-rabbit AF488 as described in Materials and Methods. Scale bar = 10 *μ*m. (b) HeLa cells were cotransfected with plasmids encoding N-Flag and HA-P jointly by lipo3000. DMSO or curcumin was added at 12 h before sample collection. At 24 h posttransfection, Cells were collected and fixed, PI4KB was stained as described above, HA-P was immunostained to visualize IBs, and nuclei were counterstained with DAPI. Scale bar = 10 *μ*m. The fluorescence intensity profile of IBs (red) and PI4KB (green) was measured along the line drawn on a zoom panel by NIS-Elements BR 4.60.00.64-bit. (c) HeLa cells were infected with HPIV3_HA-P_ at an MOI of 1.0; DMSO or curcumin was added at 12 h before sample collection. The cells were collected after 36 h infection. PI4KB and IBs were immunostained as described above, nuclei were counterstained with DAPI. Scale bar = 10 *μ*m. The fluorescence intensity profile of IBs (red) and PI4KB (green) was measured along the line drawn on a zoom panel by NIS-Elements BR 4.60.00.64-bit.

Studies have found that cellular PI4KB can interact with P protein in the HPIV3 N-P complex and be recruited and colocalized in viral IBs to help virus replication [13]. Our research in MDCK cells has found that curcumin treatment not only inhibits viral HN expression but also significantly reduces PI4KB expression in cells (Figures 5(a) and 5(b)). Moreover, upon curcumin treatment, viral HN and cellular PI4KB expression were also greatly decreased in HPIV3-infected A549 cells (Figures 5(c) and 5(d)). To further study the relationship between curcumin inhibition of PI4KB expression and HPIV3 replication, N-Flag and HA-P were coexpressed in HeLa cells, and the colocalization of endogenous PI4KB and IBs was observed by immunofluorescence after treatment with curcumin. The results indicated that under normal circumstances, endogenous PI4KB is evenly distributed in the cytoplasm and nucleus (Figure 6(a)). In the presence of N and P, PI4KB aggregates into dots and colocalized well with IBs formed by N and P. When compared with the untreated DMSO group, curcumin treatment not only affected IB formation but also seriously interfered with colocalization of PI4KB and IBs (Figure 6(b)). Similarly, in HPIV3_HA-P_-infected HeLa cells, the colocalization of PI4KB and viral IBs in cells was also greatly affected by curcumin treatment compared with the DMSO group (Figure 6(c)). These results suggest that on the one hand, curcumin downregulates PI4KB expression in cells, resulting in a decrease of PI4KB recruited into IBs, and on the other hand, it severely interferes with the colocalization of PI4KB and viral IBs, thereby affecting viral replication.

## 4. Discussion

As a natural compound, curcumin has been reported to have antiviral activity against various viruses, including the novel SARS-CoV-2 [28, 29]. Our research confirmed that curcumin could inhibit HPIV3 infection, and its antiviral effect is dose-dependent. To find the specific stage of curcumin in inhibiting HPIV3 infection, we performed three treatment conditions: pretreatment, cotreatment, and posttreatment. Studies have found that curcumin can act on the lipid bilayer envelope of the hepatitis C virus (HCV), resulting in nonlinear thinning of the lipid membrane and reducing membrane elasticity [30]. By reducing the virus membrane, curcumin interferes with HCV genome entry into the cell, thus inhibiting virus infection [31]. In the present study, curcumin has been incubated with cells before virus infection in pretreatment, while HN expression of pretreatment is comparable to that of the DMSO group, indicating that curcumin incubating with cells in advance cannot prevent HPIV3 infection. According to reports, incubating curcumin and virus can prevent virus particles from combining with host cells. For example, incubating curcumin with the influenza virus and Newcastle disease virus can destroy hemagglutinin protein (HA) on the surface of the influenza virus and HN protein on the surface of the Newcastle disease virus, which interrupts virus surface glycoprotein binding to the sialic acid receptor on the cell surface and prevents the virus from entering the host cells [22, 32]. In addition to enveloped viruses, curcumin can also prevent the nonenveloped Norwalk virus from entering cells [33]. Our study also found that incubating curcumin with HPIV3 can neutralize the virus and significantly reduce the virus' ability to infect cells (unpublished results), which may be because curcumin destroys the structure and function of glycoprotein. In cotreatment experiments, curcumin and HPIV3 are simultaneously added to cells, and the drug acts only when the virus binds and enters a host cell. Compared with DMSO treatment, viral HN expression was much lower after cotreatment. Since by 6-8 h after infection, the virus has completed the entry process, and HN expression with curcumin removal at 6 h posttreatment was lower than in cells treated with DMSO, similar to observations with other enveloped viruses, curcumin is likely to interfere with the cellular entry of HPIV3. This mechanism has not been thoroughly explored and needs to be studied further. Postinfection curcumin treatment significantly reduced HN protein expression compared with the DMSO group, indicating that curcumin is likely to also inhibit viral replication and possibly budding. However, HN expression declined with drug removal at both 6 and 12 h posttreatment, with no significant differences between cells undergoing drug removal at time points ranging from 12 to 48 h, suggesting that curcumin only affects viral replication and not budding. Of note, during virus infection, syncytia can be observed in DMSO-treated cells but not in curcumin-treated cells (Figures 3, 4, and 6). And with the extension of infection, syncytia were still not formed later (Figure 3), implying that curcumin suppresses viral infections but does not delay the viral infectious cycle.

HPIV3 replication is carried out in IBs formed by viral proteins and related host proteins in the cellular cytoplasm [8, 10, 11], since IB formation is capital for viral RNA synthesis, and viral RNAs in cells infected and co- and posttreated with curcumin were both decreased. Focusing on curcumin impact on the formation of viral IBs, our research clarified to some extent the inhibitory mechanism of curcumin on HPIV3 replication. F-actin importance to HPIV3 replication lies in that F-actin can bind to viral proteins in RNP complex or IBs and anchor it to the cytoskeleton, providing a platform to form IBs or RNPs [11]. Our research confirmed that although the cytoskeleton tubulin does not seem to be affected by curcumin, curcumin can indeed disrupt the polymerization of the F-actin filamentous structure and thus affect the formation of IBs, indicating that curcumin interrupting the F-actin skeleton is one of the key mechanisms to inhibit HPIV3 replication. In addition, the endoplasmic reticulum protein will be reconstructed and exploited during the formation of HPIV3 IBs. This process needs to rely on PI4KB-induced PI4P for lipid exchange in membrane organelles [34]. It is reported that the HPIV3 P protein interacts with PI4KB and recruits it into IBs, inducing a PI4P-enriched microenvironment, which is conducive to IB formation and facilitates viral replication [13]. In the present study, we found that curcumin treatment downregulates the PI4KB expression and interferes with the colocalization of PI4KB and IBs, thereby affecting IB formation and viral replication. Research on EV71 also found that curcumin significantly reduced the expression level of PI4KB, which is important to form a viral replication complex [23]. It implies that the antiviral mechanism of curcumin on HPIV3 and EV71 may be similar.

The structural integrity of F-actin also depends on regulating cellular PI4P [12]. Curcumin causes a decrease of PI4KB expression, resulting in a low expression of PI4P, which may indirectly affect PI4P-mediated F-actin polymerization. In other words, curcumin downregulates PI4KB and interferes with the colocalization of PI4KB and IBs, and it may also indirectly act on F-actin and influence IB formation.

Here, we identify a novel role for curcumin as an antiviral drug against HPIV3 infection through its ability to affect IB formation and viral replication. This provides a theoretical basis for research and development of HPIV3 antiviral drugs, with implications for parainfluenza virus research and other similar viruses.

## 5. Conclusions

In summary, our studies found that curcumin has a significant antiviral effect on HPIV3 infection and has varying degrees of impact on multiple stages of the viral life cycle. Curcumin can disrupt the structural integrity of F-actin, downregulate the endogenous PI4KB expression, and interfere with the colocalization of PI4KB and IBs, thereby hindering viral IB formation and inhibiting viral replication. Our findings may be a valuable target for rational antiviral approaches.

## Data Availability

The data presented in this study are available in this article and on request from the corresponding author.
